# Renal Involvement in Mixed Cryoglobulinemic Vasculitis: Current Perspectives

**DOI:** 10.3390/jcm14124369

**Published:** 2025-06-19

**Authors:** Annalisa Villa, Antonietta Gigante, Chiara Pellicano, Klara Radovic, Konstantinos Giannakakis, Milvia Casato, Marcella Visentini

**Affiliations:** 1 Department of Transitional and Precision Medicine, Sapienza University of Rome, Viale dell’Università 37, 00185 Rome, Italy; annalisa.villa@uniroma1.it (A.V.); chiara.pellicano@uniroma1.it (C.P.); klara.radovic@uniroma1.it (K.R.); milvia.casato@uniroma1.it (M.C.); marcella.visentini@uniroma1.it (M.V.); 2 Department of Molecular Medicine, Sapienza University of Rome, Viale dell’Università 37, 00185 Rome, Italy; 3 Department of Radiological, Oncological and Anatomo-Pathological Sciences, Pathological Anatomy, Sapienza University of Rome, Viale dell’Università 37, 00185 Rome, Italy; konstantinos.giannakakis@uniroma1.it

**Keywords:** mixed cryoglobulinemic vasculitis, hepatitis C, autoimmune disease, cryoglobulin, membranoproliferative glomerulonephritis, monoclonal gammopathy

## Abstract

Cryoglobulinemia is a rare disorder characterized by the presence of abnormal proteins (cryoglobulins) in the blood that precipitate at low temperatures. It presents with a wide clinical spectrum, from mild symptoms to severe, life-threatening disease. In mixed cryoglobulinemia (MC), vascular damage results from immune complexes—typically monoclonal IgM with rheumatoid factor activity and polyclonal IgG (Type II), or polyclonal/oligoclonal IgM and IgG (Type III). These complexes can obstruct small blood vessels, leading to ischemia and leukocytoclastic vasculitis. Renal involvement occurs in about 30% of MC patients and it is a manifestation with poor prognosis. Nowadays, types II and III MC are the most common forms, often linked to autoimmune diseases like Sjögren’s syndrome and systemic lupus erythematosus, or to viral infections such as hepatitis B or persisting despite hepatitis C eradication. This review explores renal involvement in MC, offering a comprehensive perspective on current knowledge, including recent advances in pathophysiological understanding, evolving diagnostic strategies, and novel therapeutic approaches. In this context, “perspectives” refers to the growing recognition of the shifting epidemiology of MC—particularly the changing etiological landscape following hepatitis C virus eradication—as well as the integration of emerging clinical and pathological entities such as cryofibrinogenemia and monoclonal gammopathy of renal significance into diagnostic and management frameworks. Furthermore, the review highlights current therapeutic strategies recognized as most effective, emphasizing the importance of a multidisciplinary and multimodal approach that combines etiological treatment, B-cell–targeted therapy (notably rituximab), plasma exchange in selected cases, and comprehensive supportive care for renal and systemic complications. Moreover, the landscape of management could be enriched in future years, since clinical trials are ongoing to explore novel therapies for refractory or relapsing cases of MC with renal involvement.

## 1. Introduction

Cryoglobulins are circulating immunoglobulins that precipitate at low temperatures and redissolve again upon rewarming. This is the classical definition of the immune complexes that are found in cryoglobulinemia, a rare pathological condition that can manifest with a wide clinical spectrum, ranging from mild symptoms to severe, life-threatening disease [1].

Organ damage is primarily due to vascular involvement through an immune-mediated mechanism, as seen in mixed cryoglobulinemia (MC) vasculitis. Here immune complexes, consisting of monoclonal IgM with rheumatoid factor activity and polyclonal IgG (type II MC), or polyclonal oligoclonal IgM and IgG (type III MC), precipitate and cause small vessels’ obstruction and ischemia, leading to leukocytoclastic vasculitis [2].

Less commonly, in type I cryoglobulinemia, often associated with hematological malignancies such as multiple myeloma, Waldenström’s macroglobulinemia, monoclonal gammopathy of undetermined significance (MGUS), or B-cell lymphoma, the vascular damage is due to hyperviscosity syndrome and the precipitating Ig consists of a single IgM or IgG.

First described in 1966 [3], the disease has progressively revealed its pathogenic mechanisms and underlying causes. In the early 1990s, hepatitis C virus (HCV) infection was identified as the leading cause of MC, redefining what were previously considered “essential” forms of the disease [4].

In 2015 the advent of direct-acting antiviral agents (DAA) led to sustained virological response and, in the vast majority of cases, to complete remission of HCV-related cryoglobulinemia [5].

Therefore, the current scenario of cryoglobulinemia has once again evolved. Beyond the rare cases of type I cryoglobulinemia, the spectrum is now predominantly composed of type II and III mixed cryoglobulinemia, most frequently in the form of truly essential cases. These are accompanied by forms associated with autoimmune disorders—such as Sjögren’s syndrome and systemic lupus erythematosus (SLE) [6]—as well as rare instances linked to hepatitis B virus (HBV) infection or persisting despite HCV eradication [7,8,9,10].

In this evolving clinical landscape, where the etiological spectrum of cryoglobulinemia has significantly shifted, kidney involvement remains one of the most serious and prognostically relevant complications. The intent of this review is to provide an updated overview of the current understanding of renal involvement in mixed cryoglobulinemic vasculitis, highlighting its pathophysiological mechanisms, diagnostic challenges, and evolving therapeutic strategies.

## 2. Epidemiology of Cryoglobulinemia and Renal Involvement

Cryoglobulinemia is considered a rare disease (prevalence of <5/10,000 in the general population in Western countries) [11]; until 2015 hepatitis C virus (HCV) was its most common cause, making cryoglobulinemia a not-so-rare disease in high endemic areas.

Cryoglobulinemia was more common in Southern Europe than in Northern Europe and North America, reflecting the higher prevalence of HCV. Among individuals infected with HCV, the rate of cryoglobulinemia diagnosis reached nearly 60% [12]. In the last decade, the use of DAA has dramatically reduced HCV prevalence and the morbidity and mortality of both liver-related and extra-hepatic manifestations of HCV disease [13,14]. A large retrospective study shows a halving of HCV-related cryoglobulinemia diagnoses in the last years (62.5% in 2011 vs. 33.3% in 2018) [15].

Nowadays, the emerging more frequent etiologies of cryoglobulinemia are becoming autoimmune diseases, mainly SLE and Sjogren’s syndrome [15].

Kidney involvement in cryoglobulinemia is an infrequent condition, affecting almost 30% of patients with cryoglobulinemia. Available data about the epidemiology of renal injury in cryoglobulinemia are extrapolated from case report and case series, with high variability in the ranges of reference. In a retrospective large cohort (*n* = 153), cryoglobulinemic glomerulonephritis (GN) was more commonly observed in type II cryoglobulinemia (65% of cases), then in type III cryoglobulinemia (28% of cases) and rarely in type I cryoglobulinemia (7%) [16].

## 3. Pathogenesis of Renal Involvement in Mixed Cryoglobulinemia

The filtration barrier including endothelium, glomerular basement membrane (GBM), and podocytes is the target of inflammatory and non-inflammatory injury in glomerular disease in general. Proximity to the circulation allows also production of anaphylatoxins and recruitment of inflammatory cells [17].

Renal involvement in the context of type II and III MC is characterized by a glomerular injury mediated by the cryoprecipitable rheumatoid factor/IgG immune complexes [11]. In particular, the disbalance between active immune complexes formation and their defective clearance, leads to subsequent organ damage [11]. Cryoglobulins deposited in renal parenchyma are able to escape the physiologic elimination provided by the reticuloendothelial system. In fact, cryoglobulins trigger complement activation and consumption, leading to an impaired ability to bind activated C3 complement component. This results in downregulation of erythrocyte complement receptor 1 (CR1), compromising erythrocyte transport and reducing their clearance by hepatic and splenic macrophages [18]. Another mechanism described in the pathogenesis of cryoglobulinemia is the fluctuation of local anion concentration, and in particular chloride anion was a strong determinant of precipitation of cryoglobulins in tissues, including kidney, independently from temperature variation [19].

In addition, a defective in situ immune system is involved in the progression of renal damage. Circulating immune complexes localize in the glomerular capillaries, but subsequently subendothelial, mesangial, and tubular cell activation leads to leukocyte infiltration and local cell proliferation.

In fact, regardless of the initial renal insult, lymphocytes and macrophages are recruited in the renal interstitium in response to chemokines and adhesion molecules. Infiltration of macrophages is detected in early phases of progressive renal disease, and it is related to the proinflammatory and profibrotic pathways. Therefore, activated macrophages induce the inflammatory milleu that bring to progressive proliferation of renal cells [20].

In cryoglobulinemic GN, in situ persistent inflammation has historically been attributed to impaired clearance of cryoglobulins, resulting from dysregulated macrophage activation. While macrophage phagocytic function seems to be preserved, there is a defect in the intracellular and lysosomal degradation of immune complexes, leading to amplification of renal injury [21].

In recent years, interest in neutrophil extracellular traps (NETs) has also focused on immune-mediated GN. NETs, released by activated neutrophils, containing granules, chromatin, histones, and autoantigens that can perpetuate inflammation. This process ultimately promotes a humoral immune response and the deposition of immune complexes, contributing to the progression of renal damage in various autoimmune diseases characterized by increased NETosis, like for example anti-neutrophil cytoplasm antibody (ANCA)—associated vasculitis and SLE. However, the precise role of NETs in renal involvement of autoimmune diseases, and especially in cryoglobulinemia, is incompletely understood [17].

In addition to the classical pathway of deposition on immune complexes within renal parenchyma, other factors may contribute to the complex pathogenesis of mixed cryoglobulinemic GN. These include in situ binding of clonally restricted IgM with high affinity for glomerular matrix components, as well as subclinical, smoldering lymphoproliferative disorders that drive chronic and progressive renal injury [11].

## 4. Clinical Manifestations of Cryoglobulinemia with Renal Involvement

Systemic symptoms of cryoglobulinemia may vary, depending on the type of cryoglobulins [22].

MC can develop with subtle manifestations, like arthralgias and asthenia. Joint involvement consists of non-migratory pain predominantly involving hands and knees in a bilateral and symmetric distribution; arthritis is less common and it is not deforming or erosive [6].

Skin involvement is common at the onset of the disease, with vascular purpura on the lower extremities being the more frequent manifestation. The lesions are infiltrated by non-pruriginous petechiae or papules, which required an appropriate differential diagnosis with infective and other autoimmune diseases [11].

The peripheral nervous system is often affected; presenting with distal sensory or sensorimotor polyneuropathy predominant at the lower limbs or mononeuritis multiplex that represents the more frequent presentations in cryoglobulinemic patients [23]. Common early symptoms include disorders in superficial sensitivity, with neuropathic pain and paresthesia.

Motor function loss is less common, delayed in the history of disease, and setting in gradually with an anterolateral and asymmetric distribution [6].

Type I cryoglobulinemia frequently causes symptoms related to thrombosis, ischemia, and/or hyperviscosity, ranging from cutaneous symptoms such as Raynaud phenomenon acrocyanosis, nonhealing lower extremity ulcers, and livedo reticularis to neurological symptoms such as blurred vision, diplopia, headache, mental status changes, and cerebrovascular events [24].

Vasculitis of the brain, although rare, is probably underestimated and can affect patients with different types of cryoglobulinemia, including manifestations such as acute/subacute neurological deficits, headaches, seizures, cranial nerve involvement, and in some cases cerebrovascular events.

The central nervous system involvement, apart from the hyperviscosity syndrome, is probably due to intravascular cryoglobulin precipitation with ischemic lesions in small brain vessels not affected from vasculitis process [25].

Cryoglobulinemic GN is a heterogenous disease with a late onset in the history of the disease; clinical manifestations are variable ranging from subclinical-mild disease to life-threatening conditions.

Renal involvement usually appears 3–5 years after the onset of skin purpuric lesions [11]. Asymptomatic mild-moderate proteinuria, with or without microscopic hematuria, is found in 30–35% of patients. However, proteinuria may reach the nephrotic range (3.5 g/24 h) in almost 20% of patients. Arterial hypertension can develop early after the appearance of the renal manifestations.

In conclusion, the most common clinical features are non-specific urinary abnormalities, such as mild-moderate proteinuria and/or microscopic hematuria, or a progressive reduction of renal function [26,27].

Other splanchnic involvement is uncommon in cryoglobulinemia, but rare cases of pulmonary, gastrointestinal, or cardiac symptoms, related to small vessel vasculitis, were reported [6,22].

## 5. Diagnostic Approach to Cryoglobulinemia

Classification criteria for cryoglobulinemia vasculitis, including cryoglobulins detection, clinical features, and laboratory finding are available [28].

However, in clinical practice, the diagnostic workup for cryoglobulinemia is often challenging. Laboratory detection of cryoglobulins requires strict adherence to warm-chain protocols, and false-negative results are possible. Therefore, when clinical suspicion for cryoglobulinemia-mediated disease remains high despite negative serum cryoglobulin findings, tissue biopsy of the affected organ, like the skin, kidney, or a peripheral nerve, can be instrumental in confirming the diagnosis of cryoglobulinemic vasculitis.

## 6. Laboratory Findings in Cryoglobulinemia

### 6.1. Cryoglobulins Detection

Laboratory tests for cryoglobulins require a precise and temperature-controlled procedure: blood samples have to be collected in tubes without anticoagulants and preserved at 37 °C until centrifugation and at least until clot formation. Then samples are stored at 4 °C to allow cryoprecipitate formation. After almost 7 days, a serum cryoglobulin concentration of more than 0.5 g/L or a cryocrit of over 0.5% to 1% is considered significant. After detection, cryoglobulin composition is determined by immunofixation or immune-electrophoresis [29].

Additional laboratory abnormalities commonly observed in cryoglobulinemia include alterations in complement components. Hypocomplementemia, particularly marked by reduced C4 levels, is a hallmark of MC and reflects ongoing complement activation and consumption. Elevated rheumatoid factor levels are typically associated with type II MC, due to the rheumatoid factor activity of monoclonal IgM. Increased levels of C-reactive protein (CRP) and erythrocyte sedimentation rate are also frequently present, serving as nonspecific markers of systemic inflammation.

The cryoglobulins detection can be associated with alterations in protein electrophoresis, ranging from hypergammaglobulinemia, linked to inflammation and rheumatoid factor positivity, to artefactual hypogammaglobulinemia, due to cryoglobulin precipitation.

### 6.2. Laboratory Features in Case of Renal Involvement

In the case of renal involvement some laboratory findings are important. 

Type II cryoglobulinemia, rather than type I or type III, is more frequently associated with renal disease. The monoclonal component is frequently IgM kappa and less commonly IgG kappa in cases of cryoglobulinemic GN [16,30]. IgG and IgM cryoglobulins are found in almost 90% of patients suffering from cryoglobulinemia, while IgA cryoglobulin are uncommon. However, IgA cryoglobulins seem to be more common in the context of cryoglobulinemia with renal disease [16]. Cryoglobulin concentration can be higher in patients with cryoglobulinemic GN.

In cryoglobulinemia, complement consumption of the C4 component is common, the decrease in both C3 and C4 is more frequent in cryoglobulinemic renal involvement. In fact, a multivariate analysis revealed that type II MC and a combined decrease in C3 and C4 levels were factors independently associated with renal involvement. Conversely, involvement of the skin and peripheral nerves has been significantly associated with cryoglobulinemia in the absence of renal disease [30].

### 6.3. The Case of Cryoglobulinemia Plus Cryofibrinogenemia

Cryofibrinogenemia is due to the presence in blood of abnormal fibrinogen that precipitated reversibly at low temperature; cryofibrinogenemia is classified as primary (essential) or secondary when associated with other conditions, such as an autoimmune diseases, infections, or malignancy [31]. Clinical manifestations of cryofibronogenemia can vary from asymptomatic to severe skin lesions, including ulcers, livedo reticularis, and gangrene [32]. Skin involvement has been attributable to thrombotic effect directly due to the presence of cryofibrinogens. Systemic thrombotic events can also occur in cryofibrinogenemia, including cerebrovascular and coronary thrombosis, thrombophlebitis, and arterial obstruction or pulmonary embolism [32]. Even if rare, renal involvement has been described in patients with cryofibrinogenemia sometimes with severe features, requiring dialytic treatment in some cases and with a fulminant course in one patient [33].

An association of cryofibrinogenemia with monoclonal gammopathies has been object of case reports, included in the concept of “monoclonal gammopathy of renal significance” [34,35].

In the case of cryoglobulinemia, an association with cryofibrinogenemia is largely elusive in clinical practice since, although the procedures of cold precipitation and immunologic characterization of the cryoprecipitate are very similar, the search for cryoglobulins is routinely performed using serum samples whereas the detection of cryofibrinogen requires the use of plasma.

A large retrospective study reported 1712 patients in which cryoproteins were simultaneously tested for cryofibrinogen (plasma samples) and cryoglobulins (serum samples). Strikingly, patients’ cryoglobulins were detected in 29% of cryofibrinogen-positive cases [36]. Cryofibrinogenia was transient or persistent and was associated with elevated CRP levels rather than with fibrinogen levels in plasma [36]. In another retrospective study, 18/29 (62%) of patients with cryoglobulinemia (97% of type II or III) also had cryofibrinogenemia; importantly, the presence of cryofibrinogenemia was associated with the need for more aggressive treatment regimens [37].

Therefore, cryofibrinogenemia should be investigated in patients with cryoglobulinemic vasculitis and prominent thrombotic features or severe manifestations including renal involvement, in order to optimize therapeutic strategy.

## 7. Histopathology of Renal Involvement in Cryoglobulinemia

Renal involvement in MC is most frequently characterized by the histological features of membranoproliferative GN. The classical histology, at light microscopy, is defined by variable grades of mesangial proliferation, duplication of GBM with cell interposition, and focal distribution of intracapillary pseudo-thrombi [26].

The pseudo-thrombi are early signs, consisting of immune complexes obstructing glomerular capillaries, usually distributed in a focal and segmental pattern. With the progression of cryoglobulin renal damage, the infiltration of leukocytes, especially monocytes/macrophages, is evident with mesangial proliferation and duplication of capillary walls [38]. In MC, necrotic and crescentic patterns are uncommon (<5% of cases), regardless of the severity of disease [39].

On the other hand, concomitant vasculitis of interlobular arteries, interstitial lympho-histiocytic reactive inflammation, and acute tubular damage can be detected.

Immunofluorescence staining varies depending on the type of circulating cryoglobulins and clonality. While in type I cryoglobulinemia, monoclonal deposits of IgG or IgM with positive staining for a monoclonal light chain are observed, usually accompanied by C1q or C3 positivity; MC typical features are deposits of both IgG and IgM with polyclonal light chains, with a dominancy of IgM and kappa light chains, together with positive staining for complement factors (C3, C4, C1q and C5b-9) [26].

Electron microscopy is essential for the diagnosis of cryoglobulinemic GN, allowing to perform differential diagnosis, especially with SLE nephropathy [17]. At electron microscopy, the typical features of cryoglobulinemic GN consist of GBM duplication with cells interposed and electron-dense deposits located more frequently in subendothelial and mesangial localization. The deposits represent the hallmark feature of cryoglobulinemic GN and present curved, microtubular, cylindrical, or annular forms, even if the localization in parenchyma is variable, increasing the risk of misdiagnosis.

In practice, when diagnostic uncertainty exists, even amorphous deposits lacking definitive substructure may support a clinicopathologic diagnosis of cryoglobulinemic glomerulonephritis—particularly when observed within a morphologic context suggestive of the disease and in the presence of clinically reported cryoglobulins. This underscores the importance of interdisciplinary collaboration and effective communication between clinicians and pathologists to enhance diagnostic accuracy.

## 8. Differential Diagnosis of Cryoglobulinemia with Renal Involvement

In the context of cryoglobulinemia with renal involvement, differential diagnosis with other hematological and autoimmune conditions is often required.

Differential diagnosis is essential and should consider clinical features and laboratory findings in order to evaluate the indication and timing of renal biopsy. In addition, when kidney biopsy is performed, histopathological differences are essential to define the correct diagnosis and to provide the best evidence-based treatment.

Monoclonal gammopathy of renal significance (MGRS) is defined as all renal disorders caused by a monoclonal protein in conditions where diagnostic criteria for multiple myeloma or other B-cell malignancies are not met, and it is usually related to MGUS [40]. As monoclonal gammopathy is common in cryoglobulinemia, some authors consider cryoglobulinemic renal involvement as a manifestation of MGRS. Peculiar histological features of MGRS can be highlighted.

MGRS can present with monoclonal immunoglobulin deposition with a variable parenchymal localization [41]. In immunotactoid glomerulonephritis and proliferative GN with monoclonal immunoglobulin deposits, only glomeruli are affected. In light-chain proximal tubulopathy, the disease is localized in proximal tubules. In immunoglobulin-related amyloidosis and monoclonal immunoglobulin deposition disease, all the renal compartments (glomeruli, tubuls, and interstitium) are involved. Immunofluorescence staining can then reveal organized or non-organized depositions; and specific analysis can define the specific immunoglobulin of the deposits, such as Congo Red for amyloidosis.

In addition, MGRS can occur also in the absence of immune complex deposition, in particular in C3 glomerulopathy and thrombotic microangiopathy.

MGRS was also described in patients without cryoglobulins’ positivity but with cryofibrinogenemia detection [34].

Cryoglobulinemia with renal involvement is typically characterized by a membranoproliferative GN, although concomitant vasculitis and interstitial and tubular involvement can occur complicating the diagnosis [39].

Clinical manifestations of MGRS vary depending on the renal involvement, ranging from progressive reduction of renal function, proteinuria, or Fanconi syndrome. In the context of atypical histological presentations, clinical and laboratory features of cryoglobulinemic vasculitis are essential in the diagnostic work-up.

Dealing with autoimmune conditions, some authors defined the entity of immune-complex glomerulonephritis as being of shared interest for both nephrologists and immuno-rheumatologists, considering cryoglobulinemia, IgA vasculitis (IgAV), lupus nephritis (LN), anti-glomerular basement membrane (anti-GBM) disease, and ANCA-GN into account [17].

IgAV is more common in childhood, but in adults renal involvement is more frequent [42]. Clinical presentation is characterized by a common cutaneous involvement with rash or purpura, arthralgia/arthritis, gastrointestinal involvement with abdominal pain or bleeding, and glomerulonephritis [43]. When indicated, renal involvement can be confirmed though renal biopsy and histopathological findings that reveal the presence of IgA, and in particular the IgA1 subclass, and complement C3 deposition in mesangial areas, together with mesangial hypercellularity detected by light microscopy [44].

LN is a frequent and severe manifestation of SLE, often presenting with massive nephrotic syndrome. The clinical presentation of SLE varies from one patient to another, including constitutional symptoms (fatigue, fever), mucocutaneous involvement (malar rash, photosensitivity), arthralgia and non-erosive arthritis, serositis (pericarditis and pleuritis), hematological abnormalities, and cardiac, neurological, and other systemic involvements [45].

LN histology is characterized by the so-called full-house staining pattern, referring to a simultaneous deposition of IgG, IgA, IgM, C3 C1q, and light chains, present in variable localizations (mesangial, endocapillary, and membranous), forming the basis for the LN classes [46].

Class II LN is characterized by mesangial proliferation, while class III LN is a vasculitis-like pattern, characterized by segmental endocapillary hypercellularity. Usually, class IV LN presents with a membranoproliferative-GN–like pattern reflected by the presence of global endocapillary hypercellularity.

Membranous GN defined as class V LN is characterized by subepithelial immune deposits, either global or segmental, which involve half or more of the capillary loops of more than half of the glomeruli together with mesangial deposits [47].

Anti-GBM, also known as Goodpasture’s disease, frequently evolutes in end-stage renal disease, with oligo-anuria and dialysis often required. Anti-GPM antibodies can be detected in the serum of patients and in kidney biopsy samples. Typically, deposition of IgG anti-GBM is a linear staining, less frequently associated with complement deposits. Anti-GBM disease by light microscopy presents with crescentic GN that can involve most glomeruli, as a consequence of acute inflammation due to autoantibodies deposition [48].

ANCA-associated vasculitis occurs with a renal involvement in the majority of cases (>80%) in microscopic polyangiitis (MPA), in 60% of cases in granulomatosis with polyangiitis (GPA), and in almost 30% of patients with eosinophilic granulomatosis with polyangiitis (EGPA).

GPA is frequently associated with cytoplasmic ANCA, antibodies to proteinase 3 (PR3) positivity. Common clinical manifestations of GPA include ear, nose, and throat (ENT) involvement with destructive nasal lesions, pulmonary nodules, and kidney involvement [49].

MPA is usually associated with perinuclear ANCA and antibodies to myeloperoxidase (MPO). MPA clinical features include rapidly progressive GN and lung involvement, often with alveolar bleeding [49].

EGPA, presents with the clinical features of asthma, eosinophilia, and peripheral neuropathy and ANCA positivity is reported in almost the 40% of patients [49].

Histological features of ANCA disease include evidence of a small-vessel vasculitis with granulomatous inflammation in MPA, with granulomas and necrosis in GPA, and with eosinophilic infiltration in EGPA. Histopathology of renal involvement is characterized by fibrinoid necrosis, proliferation of parietal epithelial cells, and eventually crescents; however, antibodies are destroyed by the inflammation process and typically kidney involvement is a “pauci-immune” GN, with scarce single immunoglobulin deposits and a variable grade of complement-positive staining [50].

A minority of patients (almost 10–15%) present with a double positivity to anti-GBM and ANCA autoantibodies, presenting a renal disease linked to a severe and relapsing disease course and often require maintenance therapy with immunosuppressors [51].

Table 1 summarized the principal differential diagnosis of MC.

## 9. Therapeutic Strategies for Mixed Cryoglobulinemia with Renal Involvement

MC with renal involvement requires a multimodal and multidisciplinary approach including: the etiological treatment of the underlying causes, to reduce the production of cryoglobulins, the immunosuppressive and anti-inflammatory treatment to reduce the immune complex formation, and the consequent inflammatory response with activation of complements and cytokines [22]. Moreover, the removal of preformed cryoglobulins to limit hyperviscosity syndrome is in some cases required. Finally, supportive care to reduce impact of comorbidities, such as arterial hypertension and diabetes mellitus and management of chronic kidney disease are also important aspects of the therapeutic strategy.

In the complex context of cryofibrinogenemia, data about treatment of cryofibrinogemia are limited and not standardized. Evidence underlines a limited effect of corticosteroids and the possible benefit of immunosuppressive treatment in secondary cryofibrinogenemia, and plasma exchange to reduce cryofibrinogens levels [32]. Although only case reports and retrospective studies are available, thrombotic manifestations and the evidence of defects in the fibrinolysis process suggest the potential role of anticoagulants and fibrinolytics in selected patients with cryofibrinogenemia [33,52].

### 9.1. Etiological Treatment for Mixed Cryoglobulinemia with Renal Involvement

As mentioned before, DAA therapy has completely revolutionized the treatment and the prognosis of HCV cryoglobulinemic vasculitis [13]. DAA therapy is the first-line choice in these patients, and sustained virological response is obtained in almost 95% of patients. With DAA therapy, complete disappearance of circulating cryoglobulins can occur in up to 50% of cases, due to the clearance of chronic antigenic stimulation provided by HCV, and it is linked to clinical improvement of vasculitis symptoms [15]. Some authors described the ability of DAA to reverse the phenotype of immune cells, specifically with the proliferation of regulatory T-cells, that are reduced in cryoglobulinemic patients before treatment and the reduction of atypical memory CD21^low^ B cells that are, in contrast, expanded in cryoglobulinemic patients before treatment [53,54,55].

Persistence of cryoglobulins possibly leading to systemic vasculitis relapse, despite viral eradication, accounts for up to 12% of cases and might need additional immunosuppressive treatments, mainly with RTX (see below) [8].

One fascinating hypothesis in disease relapse suggests that the survival of pathological B-cell clones endowed with both rheumatoid factor activity and specificity for HCV is supported, in absence of HCV, by circulating ICs and microbial or cell-derived nucleic acids that increase in events, such as bacterial infections or cancer [10,56,57].

In HBV-associated cryoglobulinemia, antiviral therapy with nucleoside analogs (Entecavir and Tenofovir) is the first-line treatment for HBV-associated cryoglobulinemia and, as in HCV-related disease, a decrease in viral replication was associated with the laboratory findings of undetectable cryocrit and normalization of rheumatoid factor [58].

Autoimmune diseases related to cryoglobulinemia have to be treated with symptomatic anti-inflammatory therapy, immunomodulatory treatments, and disease modifying anti-rheumatic therapy, depending on the activity and severity of systemic involvement, in order to achieve remission or low activity.

B-cell lymphoproliferative malignancies have to be treated with a clone-directed therapy, such as anti-CD20 rituximab (RTX) or proteasome inhibitors [59].

### 9.2. Immunosuppressive Treatment for Mixed Cryoglobulinemia with Renal Involvement

Previously, corticosteroid therapy was the first-line treatment in mixed cryoglobulinemic vasculitis, with good effectiveness in moderate–severe manifestations. However, long-term exposure, even to low–moderate doses of corticosteroids, is related to important side effects, and progressive reduction of the dose should be considered.

Nowadays, evidence is available for the role of B-cell depletion therapy, in particular RTX, as a steroid-sparing agent with efficacy on both moderate–severe manifestations and mild symptoms of MC [60].

RTX is a chimeric IgG1k monoclonal antibody RTX, directed against CD20 expressed on pre-B and mature B cells, inhibiting the proliferation of the expanded B-cell clones, responsible for producing cryoglobulins.

Renal involvement with cryoglobulinemic GN is considered a moderate to severe clinical manifestation of mixed cryoglobulinemic vasculitis, together with necrotizing skin ulcers, gastrointestinal vasculitis, and neurological manifestations.

Treatment with RTX demonstrated efficacy in renal involvement of cryoglobulinemic vasculitis, with stabilization or improvement of renal function, and improvement of active urinary sediment, with a decrease in proteinuria, was often obtained [30,61,62,63].

The overall response rate is more than 60% in cryoglobulinemic patients treated with RTX [60]. RTX retreatment has proven effective in managing relapses of MC, supporting the potential benefit of maintenance therapy in patients with severe organ involvement [64,65]. Relapses are a common risk in cryoglobulinemic vasculitis, impacting quality of life and prognosis.

The RTX hematological regimen utilized in non-Hodgkin lymphomas of 375 mg/m^2^, for four administrations weekly, is considered a high-dose regimen and it is the more frequently chosen in cryoglobulinemic vasculitis [61,63].

A high-dose regimen of RTX should be preferred in severe manifestations such as rapidly progressive GN or acute motor neuropathy, as well as in rare but life-threatening conditions, such as alveolar hemorrhage and intestinal or central nervous system vasculitis [60]. Interestingly, a therapeutic regimen with low-dose RTX (250 mg/m^2^ administered twice, weekly) demonstrated itself to be equally effective and safe in the induction of clinical remission and should be considered in moderate–severe manifestations in selected cases [64,66].

RTX is not associated with increased risk of serious adverse events compared to other immunosuppressants or high-dose glucocorticoids, presenting a good safety profile in cryoglobulinemic patients, irrespective of the etiology of the disease [60]. Caution has to be provided in case of multiple comorbidities, especially in the context of cardiovascular diseases, such as in other conditions.

HBV reactivation in the setting of RTX immunosuppression is a serious complication and can be life-threatening [67].

A complete infective screening has to be performed before RTX initiation and, in case of latent HBV infection, caution with adequate prophylactic therapy for HBV infection or periodical viral/antigenic load monitoring have to be performed.

Conversely, RTX monotherapy was not associated with increased risk of reactivation of HCV, despite the possibility of a transient elevation of viral load during treatment [63,68].

Other immunosuppressive treatment, in particular intravenous corticosteroid bolus or cyclophosphamide, should be considered in cases of refractory or highly relapsing cryoglobulinemic GN and/or other severe and life-treating manifestations, together with other supportive therapies [24].

### 9.3. Plasma Exchange in Mixed Cryoglobulinemia with Renal Involvement

Plasma exchange (or plasmapheresis) is performed in life-threatening contexts, including refractory disease, rapidly progressive GN, serious systemic vasculitis (for example, gastrointestinal vasculitis, severe mononeuritis multiplex, myocarditis extensive skin ulcers, or distal ischemia), and also in the rare condition of hyperviscosity syndrome [69]. Plasma exchange is usually used in combination with immunosuppressive treatment [6]. The effectiveness of plasma exchange is related to the rapid removal of circulating cryoglobulins, decreasing the immune complex formation and tissue injury. Currently, there are no standardized treatments protocols, but usually, two cycles of six sessions with a frequency of twice or three times a week are indicated during the intensive treatment period, possibly followed by weekly sessions. In cases undergoing rituximab therapy, plasmapheresis should be performed prior to or seven days after rituximab infusion, since it can effectively remove the therapeutic antibody [70].

### 9.4. Supportive Care and Prevention of Renal Function Decline

Blood pressure management and control of hypercholesterolemia and glycemia are fundamental in mixed cryoglobulinemic patients with kidney involvement, as in other chronic autoimmune systemic diseases, in order to mitigate cardiovascular complications and delay the progression of renal damage. In patients with stable renal function, the choice of the best medications should consider the impact on blood pressure, but also the effectiveness in reducing proteinuria, preferring renin-angiotensin system inhibitors or sodium-glucose cotransporter-2 inhibitors, in line with the most recent Kidney Disease Improving Global Outcomes (KDIGO) recommendations [71].

## 10. Prognosis and Complications in Mixed Cryoglobulinemia and Renal Involvement

The prognosis of MC is variable, depending on the etiology, the entity, and the severity of organ involvement and the coexistence of other comorbidities.

An early diagnosis with effective management strategy is fundamental to achieving the best outcome in cryoglobulinemic patients with renal involvement.

The level of circulating cryoglobulins rarely correlates with the severity of MC, probably due to the complex interaction of cryoglobulins with complement activation and the capability of the in situ formation of immune complexes [72].

Nowadays, HCV-associated cryoglobulinemia has a favorable outcome, as sustained virologic response is achieved with DAA therapy. Eradication of HCV reduces antigenic stimulation, stopping cryoglobulin production in most patients, and significantly improves symptoms and complications also in patients with renal involvement.

In patients with HCV-related cryoglobulinemia, low cryocrit was associated with immunologic response to DAA treatment, defined by undetectability of cryoglobulins and complement and rheumatoid factor normalization [73].

In a large and long-term follow-up cohort, almost the 90% of patients had a remission of cryoglobulinemic symptoms after DAA therapy (72.6% of patients had a complete response and 22.6% a partial response) and only 4.8% had no response [74]. Skin and renal manifestations were more likely to resolve with DAA than neurologic manifestations, which can be persistent due to irreversible nerve damage [75].

Apart from the risk of lymphoproliferative and hematological disorders related to type I cryoglobulinemia, the persistence of HCV infection is associated with an increased risk of developing B-cell non-Hodgkin’s lymphoma (NHL) [6].

DAA therapy, in addition to chemotherapy, showed also a benefit in disease free- survival of patients with HCV and B-cell NHL, when compared to the pre-DAA era [76].

Conversely, persistent cryoglobulinemia due to untreated chronic infections, systemic autoimmune disease with persistent activity, or untreated B-cell lymphoproliferative disorders are associated with a higher risk of systemic cryoglobulinemic vasculitis, cardiovascular complications, and also kidney involvement and progression.

Cryoglobulinemic patients with pre-existent arterial hypertension, diabetes mellitus type II, and male sex are more frequently prone to develop renal involvement [16].

Patients with cryoglobulinemic GN suffer the poorer prognosis, mostly due to high incidence of infectious diseases and cardiovascular diseases. The 10 year survival rate after a diagnosis of cryoglobulinemic vasculitis ranges from 50–90% in the case of renal involvement [72,77,78].

Prognoses vary depending on the visceral involvement, timing, and response to treatment. Male sex and renal involvement showed a bad prognosis [72]. In addition, purpura with cutaneous ulcers, older age, and high cryocrit levels were described as independent predictors of poor prognosis [79].

RTX treatment improves vasculitis control and reduces relapsing, also impacting renal outcomes [80].

In MC, the 5-year survival rate was 75% in patients with MC related to HCV, however a significant variation was observed depending on symptoms and visceral involvement. In particular, in patients with successful antiviral treatment and no visceral involvement the 5-years survival rate was almost 90%, while in patients with severe organ involvement such as renal, neurological, or multiorgan vasculitis, 5-years survival rate was lower (40–70%) [79]. Mortality increases in cases of delayed treatment and relapsing and refractory disease.

Kidney outcomes are worse in patients with rapidly progressive disease or nephrotic proteinuria at the time of diagnosis [30]. In a study, in both univariate and multivariate analysis, the isolate decrease in complement C4 levels was significantly associated with complete renal remission, while histological features of moderate–severe interstitial fibrosis and the presence of arteriolosclerosis at kidney biopsy were associated with a lack of complete renal remission [30].

## 11. New Strategies and Future Perspectives

Due to the negative prognosis in case of severe visceral involvement including the kidney and the risk of relapsing/resistant syndrome, MC is a complex disease to treat.

For this reason, different medications are being studied for severe and relapsing/resistant forms of MC, in order to optimize management and improve outcomes. Targeting complement activation, for example, with eculizumab or avacopan, already used for other inflammatory systemic diseases, is one of the strategies being studied. On the other hand, targeting B-cell activation blocking Bruton’s tyrosin kinase (BTK) or B-cell activating factor (BAFF) is another viable option with important trials ongoing.

Other immunomodulant and chemotherapy treatments have been also studied in MC.

Specifically, Bortezomib is a proteasome inhibitor approved for the treatment of multiple myeloma and other plasma cell dyscrasias; in fact, it is a well-established and effective treatment for type I cryoglobulinemia, usually associated with a monoclonal gammopathy [81]. In this context, bortezomib is both useful to control hematological disease and vasculitis symptoms, even in disease refractory to RTX therapy [82]. In MC, bortezomib’s role is less investigated. Some evidence showed a benefit of bortezomib in individual cases of MC, especially in the context of complex disease that is unresponsive to standard treatments [83,84]. However, further research, including larger clinical studies, is needed to establish its efficacy and safety profile in this context.

A case report described the successful use of hydroxychloroquine as a maintenance therapy, added to a standard-of-care regimen based on rituximab and plasma exchange, in the complex setting of a relapsing cryoglobulinemic vasculitis with renal involvement after eradication of HCV, with a progressive improvement in renal function until normalization in two years [85].

BTK has a crucial role in B-cell activation and proliferation. BTK inhibitors are used in Waldenstrom macroglobulinemia, where the somatic mutation in MyD88 triggers activation of the NF-κB pathway via BTK. Three case reports suggested the role of BTK inhibition with ibrutinib in mixed cryoglobulinemic patients with refractory disease, both in the context of Waldenstrom macrogloglobulinemia suspicion with MyD88 mutation and in essential cryoglobulinemia without MyD88 mutation; however, further studies are needed to confirm this evidence [86,87,88].

Eculizumab is a monoclonal antibody targeting the complement C5 component and preventing the proinflammatory effects of complement activation. Eculizumab is approved for paroxysmal nocturnal hemoglobinuria, being effective to reduce intravascular hemolysis.

The role of eculizumab in renal involvement from different causes has been explored [89].

In one small one-arm uncontrolled multicentric trial, eculizumab in primary membranoproliferative GN, despite an effective complement inhibition, failed to reduce proteinuria in a complete and persistent way [90].

One study exploring the role of eculizumab in a patient with MC with renal involvement and consistent neutrophilic infiltrate at renal biopsy, in addition to immunosuppressive treatment after plasma exchange interruption, with the objective of stabilizing or improving renal function, failed to achieve the goal and was stopped [91].

For these reasons, no conclusive evidence supports the role of complement inhibition in cryoglobulinemia and further research is needed.

Avacopan is a small molecule C5a receptor antagonist, not interfering with the formation of the membrane attack complex, central for the clearance of encapsulated bacteria [92]. Avacopan is approved in addition to immunosuppressive treatment in ANCA-associated vasculitis, as a steroid sparing therapy [92,93,94,95]. In particular, in the subgroup of patients with ANCA-associated vasculitis and renal involvement, avacopan showed improvement in kidney function in patients treated with immunosuppressors and permitted the reduction of steroid exposure [93,94]. Complement activation is a well-established feature of small vessel vasculitis pathogenesis. In particular, the alternative complement pathway is a key factor of autoimmune dysregulation associated with ANCA-associated vasculitis; conversely, in MC the specific role of the C5a pathway is not so clearly established [96]. There might be a theoretical rationale for exploring complement inhibitors like avacopan in cryoglobulinemia; however, clinical trials are currently lacking for this disease.

Anti-BAFF treatment, belimumab, in association with rituximab in refractory/relapsing MC, showed promising results in a pilot study in four patients, resulting in a positive impact both on systemic vasculitis symptoms and nephropathy [97]. The TRIBECA study, a double blind multicentric randomized controlled phase 2 trial, is actually ongoing to confirm the effectiveness of belimumab added to the standard of care in active cryoglobulinemic vasculitis [98].

In summary, the future of managing renal involvement in MC, especially in the context of refractory/relapsing disease, appears promising with ongoing research focusing on innovative therapeutic approaches. Specifically, early findings suggest a potential role for BTK inhibitors, needing further studies for validation in larger cohorts. Concurrently, anti-BAFF agents like belimumab, undergoing advanced evaluation, represent a novel promising strategy to improve patients’ outcomes.

Given the complexity of MC, a personalized treatment approach is essential.

**Figure 1:**
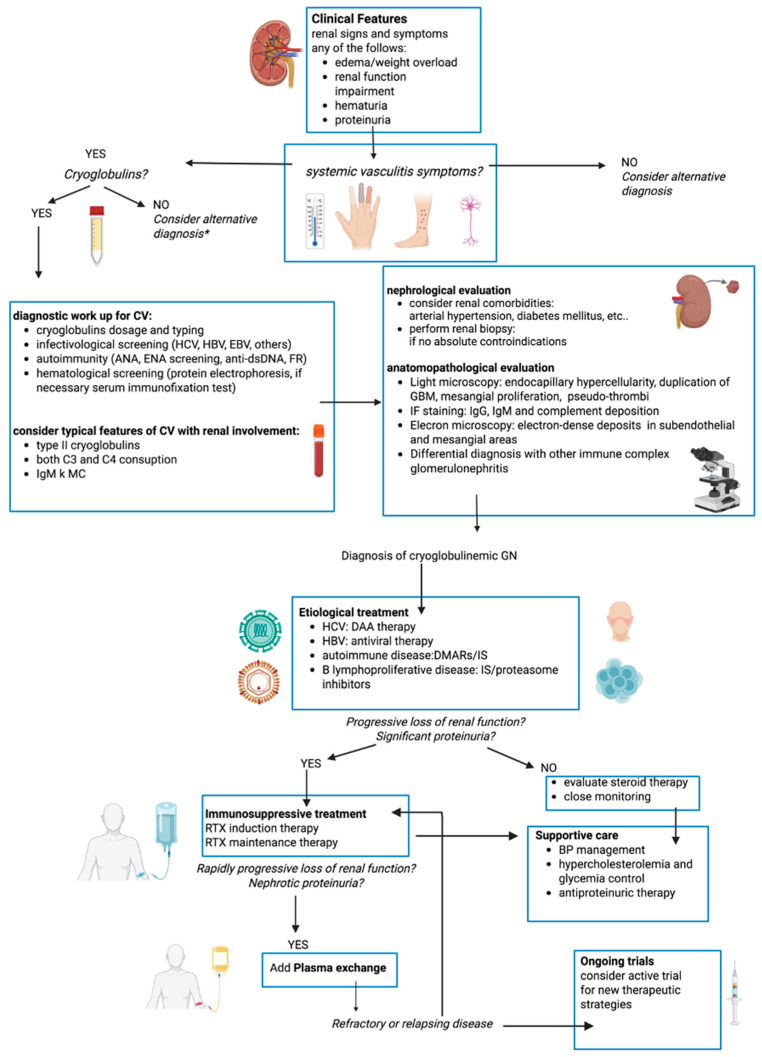
Flow chart of diagnostic-therapeutic work-up of mixed cryoglobulinemic vasculitis with renal involvement. CV: cryoglobulinemic vasculitis, HCV: hepatitis C virus, HBV: hepatitis B virus, HIV: Human Immunodeficiency Virus, GBM: glomerular basement membrane, RTX: rituximab, DAA: direct-acting antiviral agents, DMARs: Disease-Modifying Antirheumatic Drugs, IS: immunosuppressors. * but if high suspect consider cryofibrinogenemia and repeat cryglobulins dosage periodically.

**Figure 2:**
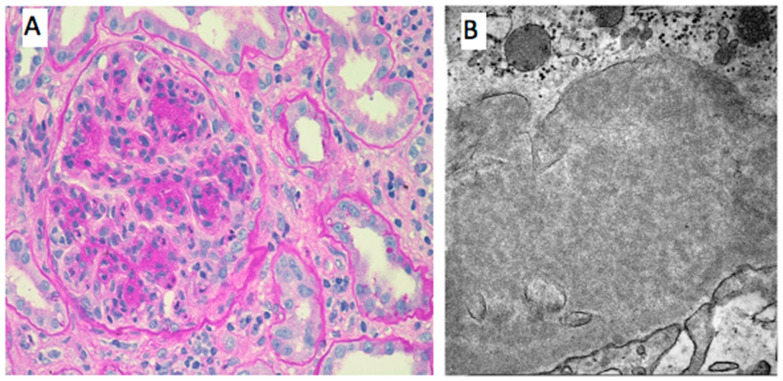
Histological features of renal involvement in cryoglobulinemic vasculitis. (**A**). PAS 400×-Lobular aspect of the glomeruli due to mesangial and endocapillary proliferation. Glomerular capillary walls are thickened by duplication of glomerular basement membrane and mesangial interposition. Focal intracapillary leucocytic infiltration. (**B**). EM 8000×-Microtubular aspect of the subendothelial cryoglobulin deposits.

Future perspectives include the identification of specific biomarkers, such as immunological, inflammation markers, or microRNAs [99,100] to better characterize patients, optimize management, and improve outcomes (Figure 1 and Figure 2).

## Figures and Tables

**Table 1:** Principal differential diagnosis of Mixed Cryoglobulinemia.

Disease	Extrarenal Manifestations	Renal Clinical Features	Laboratory Findings	Kidney Histopathology	Therapy
Mixed Cryoglobulinemia	Fatigue, purpura, arthralgia, peripheral neuropathy	Proteinuria (no neprhotic range), hematuria, loss of renal function	Positive cryoglobulins, low C4, rheumatoid factor, possible HCV positivity	Membranoproliferative GN; immune complex deposition; GBM duplication and electron-dense deposits	Treat underlying cause (e.g., antivirals for HCV), immunosuppressants, rituximab for severe cases
MGRS	Often asyntomatic; if symptomatic systemic aspecific symptoms (fever, malaise, asthenia)	Proteinuria, Fanconi syndrome	Monoclonal protein, no multiple myeloma criteria	Monoclonal immunoglobulin deposits; variable localization (glomeruli, tubules, interstitium); Congo red+ for amyloidosis	Target monoclonal clone (e.g., with chemotherapy); supportive renal care
IgA Vasculitis	Cutaneous purpura, arthralgia, GI symptoms	Hematuria, proteinuria	IgA1 and C3 in serum/urine, often mild systemic inflammation	Mesangial IgA1 and C3 deposition; mesangial hypercellularity	IS (e.g., steroids), supportive care
Lupus Nephritis	Systemic lupus symptoms: rash, arthritis, nephrotic syndrome	Proteinuria in nephrotic range	ANA, anti-dsDNA, low C3/C4	Full-house staining (IgG, IgA, IgM, C3, C1q); proliferative or membranous patterns	IS (e.g., corticosteroids, MMF, CYC); class-dependent
Anti-GBM Disease	Systemic symptoms like fever, malaise, and joint pain, rarely neurological issues	Oligo-anuria, severe renal failure	Anti-GBM antibodies, possible ANCA co-positivity	Linear IgG staining; crescentic GN	Plasmapheresis, steroids, IS (e.g., cyclophosphamide)
ANCA-Associated Vasculitis	ENT/pulmonary (GPA), lung bleeding (MPA), asthma/eosinophilia (EGPA)	Rapidly progressive loss of renal function	ANCA (PR3 or MPO), eosinophilia (EGPA), elevated inflammatory markers	Pauci-immune GN; crescents, fibrinoid necrosis, granulomas (GPA/MPA), eosinophils (EGPA)	IS (e.g., steroids, rituximab, CYC); maintenance therapy in relapsing forms

MGRS: monoclonal gammopathy of renal significance, HCV: hepatitis C virus, GN: glomerulonephritis, GI: gastrointestinal, ENT: Ear, Nose, and Throat, GBM: Glomerular Basement Membrane, GPA: Glomerular Basement Membrane, EGPA: Eosinophilic Granulomatosis with Polyangiitis, MPA: Microscopic Polyangiitis, PR3: Proteinase 3, MPO: myeloperoxidase, ANCA: Antineutrophil Cytoplasmic Antibody, IS: immunosuppressors.

## Data Availability

Non applicable.

## Abbreviations

mixed cryoglobulinemia (MC), monoclonal gammopathy of undetermined significance (MGUS), hepatitis C virus (HCV), direct-acting antiviral agents (DAA), systemic lupus erythematosus (SLE), hepatitis B virus, GN = glomerulonephritis (HBV), anti- neutrophil cytoplasm-antibody glomerulonephritis (ANCA), IgA vasculitis (IgAV), lupus nephritis (LN), anti- anti-glomerular basement membrane (anti-GBM), microscopic polyangiitis (MPA), granulomatosis with polyangiitis (GPA), eosinophilic granulomatosis with polyangiitis (EGPA), neutrophil extracellular traps (NETs), monoclonal gammopathy of renal significance (MGRS), rituximab (RTX).
